# Influence of perceived importance of the internet on life satisfaction and health of the older people: An analysis based on intermediary and moderating effects

**DOI:** 10.3389/fpubh.2022.952619

**Published:** 2022-08-19

**Authors:** Kai Gao, Mao-min Jiang, Zheng-yu Wu, Pei-pei Guo

**Affiliations:** ^1^School of Management, Shanghai University of Engineering Sciences, Shanghai, China; ^2^School of Public Affairs, Xiamen University, Xiamen, China

**Keywords:** family atmosphere, health status, structural equation model, perceived importance of the internet, life satisfaction

## Abstract

With the global growth of the aging population, healthy aging and active aging has become an important goal for the future social development of all countries. The purpose of this study is to explore the potential relationships between the older people's perceived importance of the Internet, family atmosphere, behavioral independence, life satisfaction, and health. The data come from the China Family Panel Studies' fourth wave (2015–2016) and fifth wave (2017–2018) investigations. According to an analysis of data of 5,948 people over 60 years old performed using LISREL 8.8 software, the selected cases answered the same questions about the perceived importance of the Internet, life satisfaction, and health status in two waves of surveys. The results show that life satisfaction and self-rated health have cross influences, while at the same time both are persistent in the time baseline, and family atmosphere and behavioral independence play an important intermediary role. Therefore, strengthening parent–child interaction, promoting parent–child relationships, and improving behavioral independence can effectively improve the life satisfaction and health status of the older people.

## Introduction

The number and proportion of older people aged 60 and above are increasing worldwide. According to public data from the World Health Organization, the global population aged 60 and above was one billion in 2019 but is predicted to increase to 1.4 billion in 2030 and 2.1 billion in 2050 (1). Against the background of global aging, caring for older people is an indispensable and urgent social governance goal of all countries worldwide (2). According to data released by the National Bureau of Statistics of China in 2020, the number of older people aged 60 and above in China reached 264 million, accounting for 18.70% of the total population, of which 161 million were aged 65 and above, accounting for 13.50% of the total population (3). Some experts predict that the number of people aged 65 and above may rise to 330 million by 2050, accounting for about a quarter of the total population (4). China is rapidly becoming an aging society. Due to the large number of older people, the Chinese government attaches great importance to their health problems, and vigorously advocates the strategy of healthy and active aging, aiming to create a favorable social environment for the upcoming severe situation of an aging population in China.

Internet information is disseminated all over the world, and China accounts for a significant amount of global Internet use (5, 55). In terms of use of the Internet, the older people represent a vulnerable group. With the development of society, paperless offices, digital banking, travel health codes, and so on all require use of network devices (6); therefore, exploring perceptions of the Internet among the older people is particularly important for enhancing the life satisfaction of this group (7). Studies have found that the higher the frequency of Internet use, the higher the individual's life satisfaction (8, 9). Some studies have also pointed out that moderate Internet use can help people temporarily overcome negative emotions, so that their negative emotions can be transferred or even reduced, thus achieving the effect of relieving anxiety and depression and promoting physical and mental health. Online communication with friends, relatives, and even strangers through the Internet is helpful to vent personal feelings, greatly dispel negative emotions, and has a lasting effect (10). A study pointed out that the Internet can provide users with rich and colorful instant information, and its impact on people's health is becoming increasingly important (11). For example, the health knowledge at a person's fingertips on the Internet can provide answers to immediate health needs for different groups of people, greatly improve an individual's health literacy (12), and positively influence their health-care service needs (13), thus promoting individual health management (14). Therefore, it is worth exploring whether perceived importance of the Internet impacts the life satisfaction and health of the older population.

Family atmosphere refers to the environment created by the behavior or language used among family members (54), such as *via* communication and interpersonal relationships (15). Communication and dialogue between parents and children can significantly enhance the emotional depth between them and create a positive, warm atmosphere in the home. Through successful interactions, misunderstanding and dissatisfaction will be minimized and harmonious coexistence between parents and children will be promoted, thereby improving the happiness of family members (16). Some studies have pointed out that the more times a person communicates with their parents and the more family activities are undertaken, the more harmonious the parent–child relationship will become. Hence, the parent–child relationship is positively related to the life satisfaction of family members. A good relationship helps to improve family members' life satisfaction (17). Some scholars have also pointed out that the intergenerational interaction between parents and children has a positive correlation with their satisfaction with the parent–child relationship, and this will positively affect their satisfaction with family life (18). The dialogue and communication between parents and children, and humanitarian care among families can not only promote the establishment of a harmonious parent–child relationship for the older people, but also help them to have a healthy physical and mental state and reduce the risk of depression (19) and chronic diseases (20). At the same time, mutual health care among family members also helps to promote the physical activity of the older people and makes them more willing to go out to participate in healthy activities in the process of communication with family members, so as to maintain a healthy physical condition (21) and reduce the risk of chronic diseases (22). For example, the “filial piety culture” strongly advocated in China aims to build a harmonious and positive family atmosphere, encouraging family members to help each other and strengthening communication and exchange. This can not only improve the family atmosphere, but also enhance the happiness and physical and mental health of family members (23). However, there is a need to further examine whether family atmosphere influences the relationship between perceived importance of the Internet and life satisfaction and health of older people through a mediating effect.

Behavioral independence is a basic strength of individuals with respect to the quality of their daily life. It refers to not being attached or subordinated to a relationship, and relying on one's own strength to complete tasks. In terms of ideology, it refers to having independent thoughts, an independent personality, and the ability to live alone (52). The older people become older, their ability to act independently also declines. In rehabilitation medicine, behavioral independence is the most basic ability of people at home and in the community. If it is damaged or reduced, this will directly limit self-caring ability and affect connection with others and society (24). Therefore, in the opinion of most experts and scholars, behavioral independence is very important for the older people, and it is one of the important indicators reflecting their quality of life (25). The existing research shows that older people with good behavioral independence are usually willing to take an active part in community activities and housework. Their mental state will be improved accordingly due to their physical activity. Their depression will be obviously lower, the threshold of dysthymia will be relatively high (26), and they can have higher subjective well-being (27). Moreover, older people with better independent behavioral ability generally have better nutrient absorption capability and are more willing to actively participate in physical or intellectual work, which will greatly reduce their risk of Alzheimer's disease (28). Overall, older people's perceptions of the importance of the Internet can promote their life satisfaction and self-rated health independently and through family atmosphere and behavior—the latter of which needs to be further verified with reference to national survey data.

**Figure 1:**
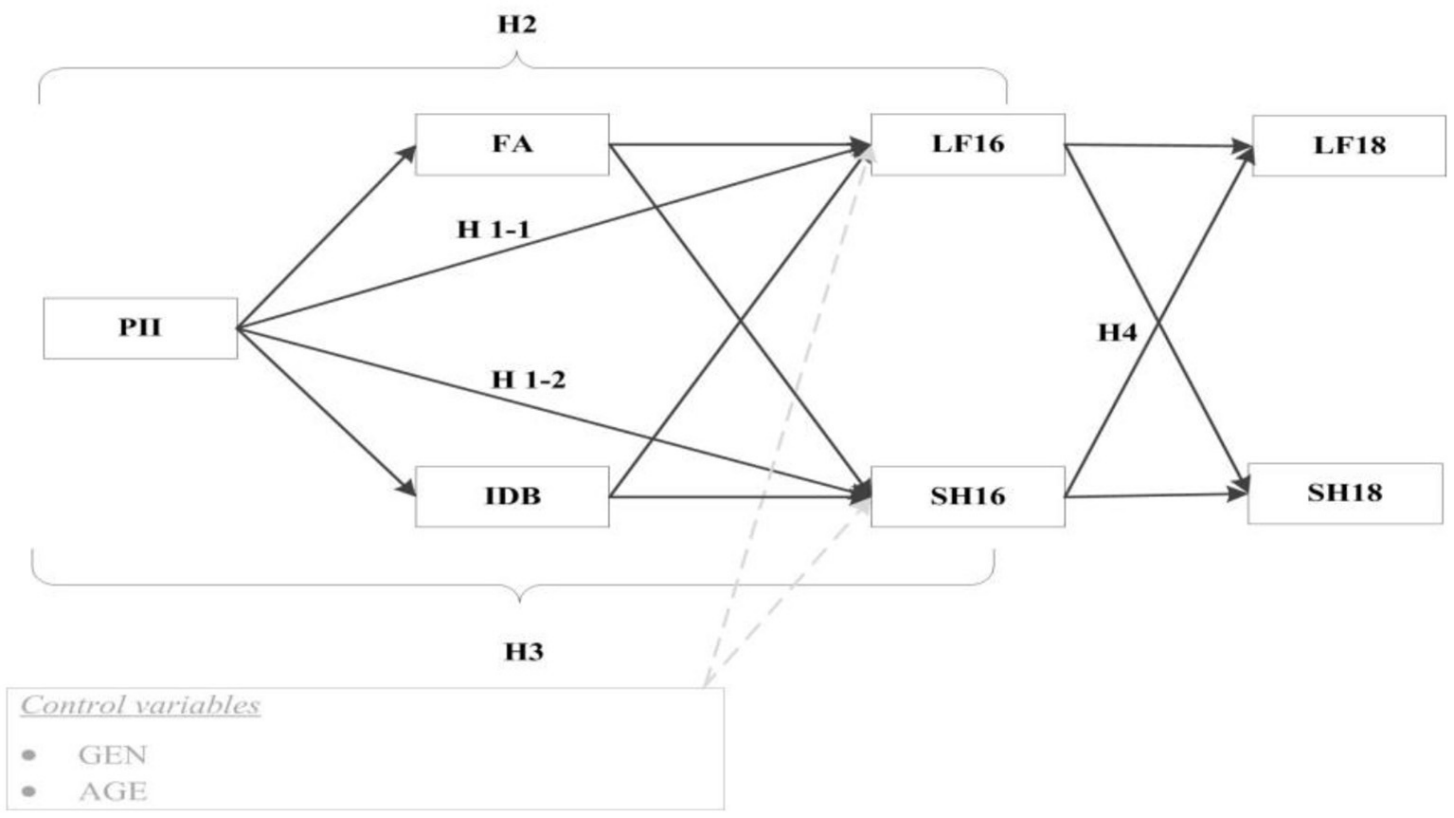
Hypothesized model of the research framework. PII, perceived importance of the Internet; FA, family atmosphere; IDB, independent behavior; LF16, life satisfaction in 2015–2016; SH16, self-rated health in 2015–2016; LF18, life satisfaction in 2017–2018; SH18, self-rated health in 2017–2018.

What is the relationship between the perceived importance of the Internet, life satisfaction, and self-rated health of older people? Are family atmosphere and behavioral independence mediating factors between perceived importance of the Internet and life satisfaction/self-rated health? Did life satisfaction and self-rated health in 2016 affect life satisfaction and self-rated health in 2018? There has been little research on the impact path between perceived importance of the Internet and the life satisfaction and health of the older people. Therefore, based on existing theories and literature, several research hypotheses are shown (see Figure 1). These are as follows: H1-1) The importance of the Internet as perceived by the older people will have a positive impact on life satisfaction in 2016; H1-2) The importance of the Internet as perceived by the older people will have a positive impact on self-rated health in 2016; H2, H3) Family atmosphere and behavioral independence play a potential mediating role in the relationship between perceived importance of the Internet and life satisfaction and self-rated health; H4) There will have a persistent cross effect between life satisfaction and self-rated health.

## Materials and methods

### Participants

The data was obtained from the fourth wave (2016) and fifth wave (2018) surveys of the China Family Panel Studies (CFPS), which is a nationally representative, biennial longitudinal survey of Chinese communities, families, and individuals launched in 2010 by the Institute of Social Science Survey of Peking University, China. The CFPS is designed to collect individual-, family-, and community-level longitudinal data in contemporary China. The research performed a baseline survey in 25 provinces, municipalities, and autonomous regions in China, and finally completed interviews with 14,960 households and 42,590 individuals, which were conducted every 2 years. A total of 36,892 people were surveyed in the fourth wave (2016) and 37,354 in the fifth wave (2018). The selection process of the research samples is shown in Figure 1. A total of 36,892 people participated in the fourth wave of the survey. First, 8,149 individuals that did not participate in the fifth wave of surveys were excluded. Second, 21,482 people younger than 60 years old were excluded. Finally, 1,313 individuals with a large number of blank questionnaires were excluded. Thus, the final sample comprised 5,948 people.

### Measures

#### Perceived importance of the network

Perceived importance of the Internet was measured by two indicators, including “the importance of the Internet as an information channel” and “the importance of Mobile phone short message as an information channel.” A 5-point Likert scale was used, ranging from 1 (very unimportant) to 5 (very important). The higher the total score, the more important the Internet was considered to be.

#### Life satisfaction

In the fourth and fifth waves, life satisfaction was measured by asking participants about their own life satisfaction, A 5-point Likert scale was used, ranging from 1 (very dissatisfied) to 5 (very satisfied). The higher the total score, the more satisfied the older people were with their lives.

#### Self-rated of health

Self-rated of health was measured by asking the participants, “What do you think of your health?” A 5-point Likert scale was used, ranging from 1 (unhealthy) to 5 (very healthy). The higher the total score, the healthier the individual considered themselves to be.

#### Family atmosphere

Family atmosphere included two aspects: parent–child relationship and parent–child interaction. The parent–child relationship was measured by asking the respondents, “How is the relationship with your children?” A 5-point Likert scale was used, ranging from 1 (not very close) to 5 (very close). Parent–child interaction was mainly defined by asking questions about contact frequency with children and meeting frequency with children. A 5-point Likert scale was used, ranging from 1 (never) to 5 (every day). The higher the total score, the higher the level of parent–child interaction.

#### Behavioral independence

This study used seven items to measure the behavioral independence of the older people, including whether they can use public transportation independently, whether they can perform independent kitchen activities, whether they can do outdoor activities independently, whether they can do laundry independently, whether they can do cleaning independently, whether they can shop independently, and whether they can eat independently. Participants described their situation according to the questions (0 = no, 1 = yes). The Cronbach's alpha value for independent behavior in the present study is 0.87.

### Data analysis

**Figure 2:**
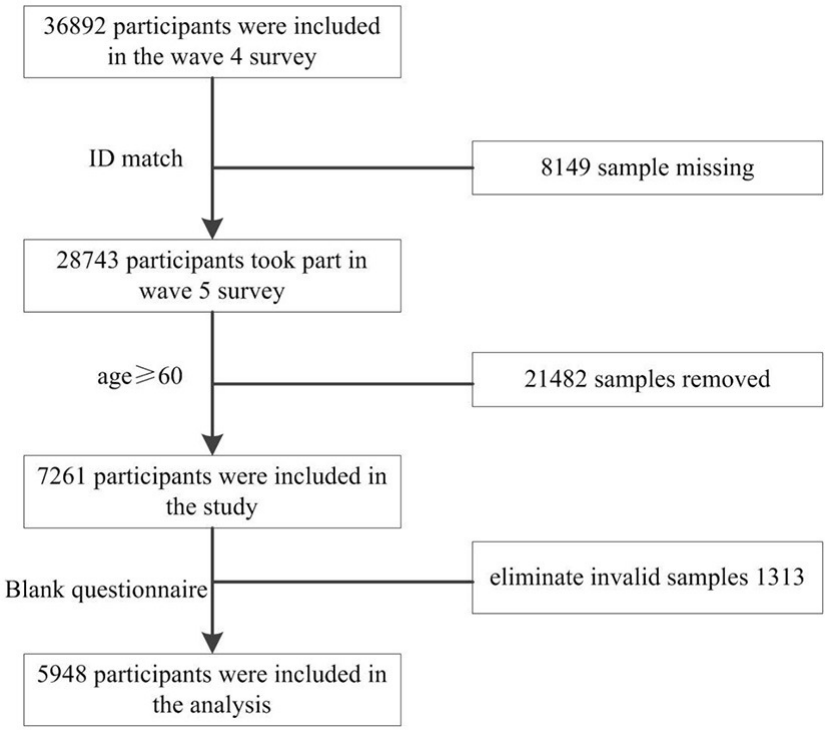
Flow chart depicting participant inclusion and exclusion.

The distributions of background characteristics and independent, mediating, and dependent variables were expressed in terms of the frequency distribution, mean, maximum, and minimum. Structural equation modeling was used to test the mediation effect. The mediating variables were family atmosphere and behavioral independence. The independent variable was the perceived the importance of the Internet and the dependent variable was sense of safety and self-rated health (Figure 2). The incremental fit index (IFI), comparative fit index (CFI), non-normed fit index (NNFI), adjusted goodness-of-fit index (AGFI), and normal fit index (NFI) indicate a good fit to the data when values exceed 0.90 (29, 30). The root mean squared error of approximation (RMSEA) value of <0.05 indicated a “close fit” (31, 32). Additionally, Bollen's relative fit index (RFI) measures the discrepancy between the model evaluated and the baseline model, indicating good model–data fit for values close to 1 (33). A Hoelter's Critical N (CN) of 200 or better indicated a satisfactory model–data fit (34). The effects specified were estimated by means of the maximum likelihood method to estimate the polyserial, polychoric, and product–moment correlations by programming in PRELIS2 (35). Statistical analyses were conducted using SPSS 22 and LISREL 8.8 statistical software.

## Results

### Descriptive data

Table 1 shows the main demographic characteristics and descriptive statistics of the respondent variables. Among the 5,948 participants, 3,033 were male and 2,915 female, and their ages were mainly between 60 and 69 years old. The average score of perceived importance of the Internet was 1.42 (SD = 0.82). Among the variables of family atmosphere, the average score of parent–child interaction was 3.26 (SD = 1.51). In response to questions about the parent–child relationship, 39.00% of respondents said close and 43.56% very close. The average score of life satisfaction in 2016 was 3.87 (SD = 1.05), and the score of life satisfaction in 2018 was 4.25 (SD = 0.92). In 2016, 26.78% rated their health as being unhealthy, 23.69% as average, 32.09% as relatively healthy, 10.57% as healthy, and 6.86% as very healthy. In 2018, 29.86% rated their health as being unhealthy, 16.96% as average, 36.45% as relatively healthy, 8.98% as healthy, and 7.75% as very healthy.

**Table 1:** Descriptive statistics variables of the sample (*n* = 5,948).

**Variable**	***n* ^#^**	**%**	**Mean**	**SD**
**Control variable**				
**Sex**				
Male	3,033	50.99		
Female	2,915	49.01		
**Age**				
60–69	4,174	70.17		
70–79	1,537	25.84		
≥80	237	3.98		
**Independent variable**				
Perceived importance of the Internet [1–5]			1.42	0.82
**Mediating variable**				
**Family atmosphere**				
Parent–child interaction [1–5]			3.26	1.51
**Parent–child relationship**				
Very bad	27	0.49		
Bad	55	0.99		
Common	882	15.96		
Better	2,156	39.00		
Very good	2,408	43.56		
Independent behavior [0–1]			0.94	0.16
**Dependent variable**				
Life satisfaction in 2016 [1–5]			3.87	1.05
**Self-rated health in 2016**				
Unhealthy	1,593	26.78		
average	1,409	23.69		
relatively healthy	1,909	32.09		
healthy	629	10.57		
Very healthy	408	6.86		
Life satisfaction in 2018 [1–5]			4.25	0.92
**Self-rated health in 2018**				
Unhealthy	1,776	29.86		
average	1,009	16.96		
relatively healthy	2,168	36.45		
healthy	534	8.98		
Very healthy	461	7.75		

*Total number < n = 5,948 due to missing*.

*[1–5], [1–0]: The range of a single item*.

### Mediation analyses

**Figure 3:**
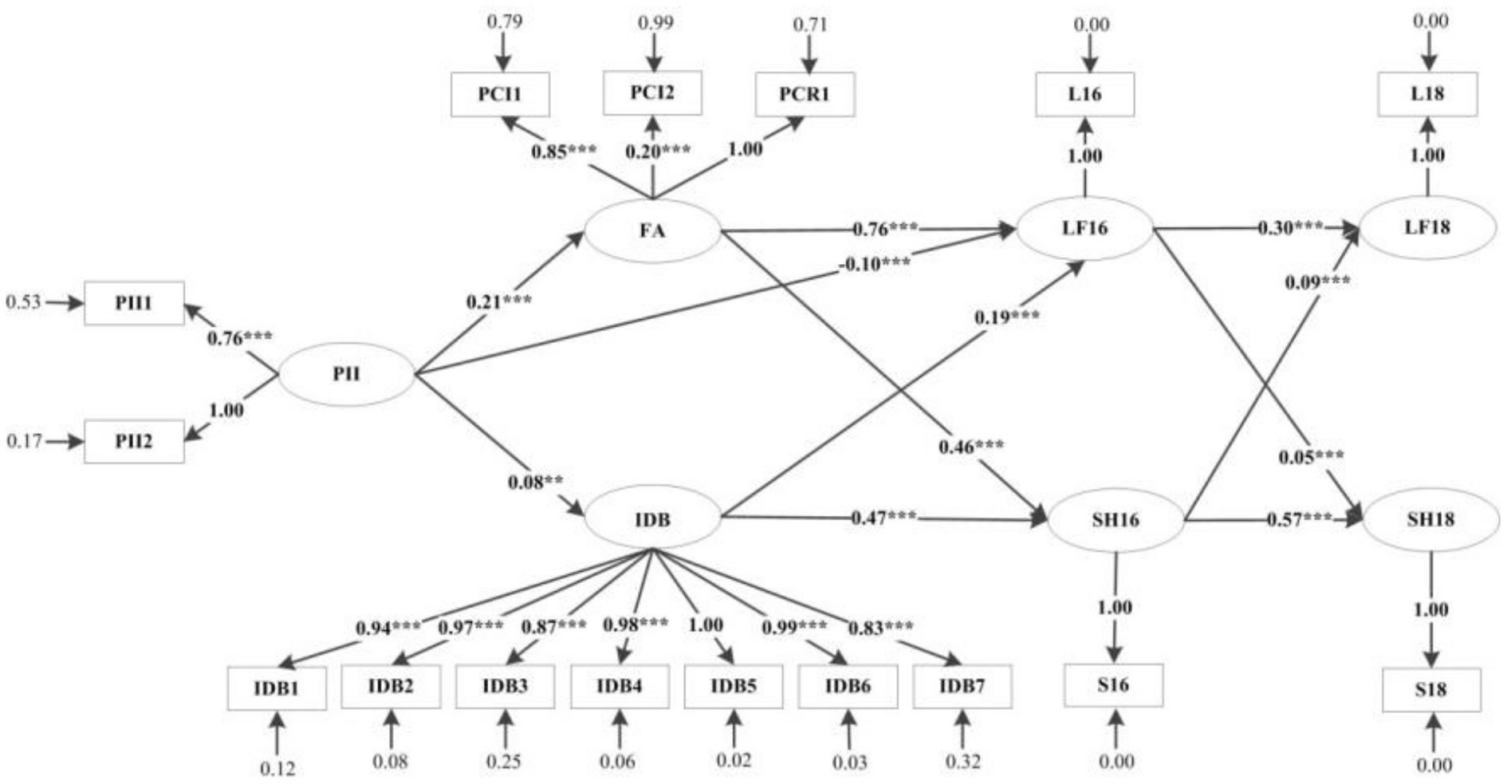
Structural equation modeling results. PII, perceived importance of the Internet; FA, family atmosphere; IDB, independent behavior; LF16, life satisfaction in 2015–2016; SH16, self-rated health in 2015–2016; LF18, life satisfaction in 2017–2018; SH18, self-rated health in 2017–2018; **p < 0.01, ***p < 0.001.

Using structural equation modeling (SEM), we determined the *p*-value (*p* > 0.05). Based on these, the insignificant path was removed from the model, and the final model was obtained. Compared with the original model, the final model's degree of fit improved to some extent, with RMSEA = 0.037, NNFI = 0.95, CFI = 0.96, IFI = 0.96, AGFI = 0.99, and CN = 857.20 (Table 2). The final model is shown in Figure 3. Findings show that older people's perception of the importance of the Internet has a significant positive impact on family atmosphere and behavioral independence (*p* < 0.05). It had a significant negative impact on life satisfaction in 2016 (*p* < 0.05), but had no significant correlation with self-rated health in 2016. Family atmosphere and behavioral independence were significantly positively correlated with life satisfaction and self-rated health in 2016 (*p* < 0.05). At the same time, life satisfaction and self-rated health in 2016 were both positively significantly related to life satisfaction and self-rated health in 2018 (*p* < 0.05).

**Table 2:** Measures of goodness-of-fit for perceived the importance of the Internet, life satisfaction, and self-rated health model of the older people.

**Model**	**RMSEA**	**NNFI**	**CFI**	**IFI**	**AGFI**	**CN**
Initial model	0.037	0.95	0.96	0.96	0.99	847.23
Delete PII → SH	0.037	0.95	0.96	0.96	0.99	853.02
Delete GEN → LF16 #	0.037	0.95	0.96	0.96	0.99	857.20

*PII, perceived importance of the Internet; SH16, self-rated health of the older people in 2016; GEN, gender; LF16, life satisfaction of the older people in 2016*.

# *Goodness-of-fit of the final model*.

First, Table 3 lists the paths from the older people's perception of the importance of the Internet to their life satisfaction and self-rated health. We found that the perceived importance of the Internet has a direct positive effect on family atmosphere (β = 0.21, *p* < 0.001), and a significant positive effect on behavioral independence (β = 0.08, *p* < 0.05) (Figure 3). We did not find that it has a direct impact on the self-rated health of the older people. At the same time, we found that the total effect of the perceived importance of the Internet and life satisfaction was 0.08 (*p* < 0.001), and the direct effect was negative (β = −0.10, *p* < 0.001), indicating that the perceived importance of the Internet can positively affect life satisfaction through intermediary variables. Therefore, H1 is partially supported.

**Table 3:** Direct and indirect effects of the older people's perceived importance of the Internet on life satisfaction and self-rated of health.

**Variables**	**Life satisfaction in 2016**			**Self-rated health in 2016**			**Life satisfaction in 2018**			**Self-rated health in 2018**		
	**Direct effect**	**Indirect effect**	**Total effect**	**Direct effect**	**Indirect effect**	**Total effect**	**Direct effect**	**Indirect effect**	**Total effect**	**Direct effect**	**Indirect effect**	**Total effect**
Perceived importance of the Internet	−0.10^***^	0.18^***^	0.08^***^	—	0.14^***^	0.14^***^	—	0.04^***^	0.04^***^	—	0.08^***^	0.08^***^
Family atmosphere	0.76^***^	—	0.76^***^	0.46^***^	—	0.46^***^	—	0.27^***^	0.27^***^	—	0.30^***^	0.30^***^
Independent behavior	0.19^***^	—	0.19^***^	0.47^***^	—	0.47^***^	—	0.10^***^	0.10^***^	—	0.28^***^	0.28^***^

** *p < 0.01*,

*** *p < 0.001*.

Second, the model shows that family atmosphere has a direct impact on life satisfaction and self-rated health, among which family atmosphere positively affects life satisfaction (β = 0.76, *p* < 0.001), and the direct impact on self-rated health is 0.46 (*p* < 0.001). Behavioral independence also has a direct impact on life satisfaction and self-rated health: the higher the behavioral independence of the older people, the better their life satisfaction and health. Therefore, H2 and H3 are supported to some extent.

Finally, the study found that the older people's life satisfaction and self-rated health have a continuous cross effect, specifically, the life satisfaction of the older people in 2016 has a significant positive impact on their life satisfaction and self-rated health in 2018. The higher the life satisfaction of the older people in 2016, the higher it is in 2018 (β = 0.30, *p* < 0.001), and the healthier the older people in 2018 (β = 0.05, *p* < 0.001). In addition, the healthier the older people in 2016, the higher their life satisfaction (β = 0.09, *p* < 0.001) scores and self-rated health (β = 0.57, *p* < 0.001) scores in 2018. Therefore, H4 is supported.

## Discussion

This study uses an intermediary model to test the influence of the perceived importance of the Internet on the life satisfaction and self-rated health of the older people. The results show that the perceived importance of the Internet can positively affect the life satisfaction and self-rated health of the older people through family atmosphere and behavioral independence. At the same time, this study also found that there is a persistent cross effect between life satisfaction and self-rated health of the older people.

With the development of communication technology, human society has entered the digital era (36, 37). For the older people, the higher their perception of the importance of the Internet, the more they will use network devices. Communication equipment is a tool and means of digital communication (38). The older people can talk with their children by using mobile phones or PCs. Studies have shown that effective communication can promote family harmony (39), while a good family atmosphere can promote personal physical and mental health (40). Family atmosphere plays an important role in the life of the older people. Harmonious family relationships and full family interaction can optimize the health management services of the older people, reduce their depression and the risk of illness and also help to improve their life satisfaction (41–43). Therefore, in order to improve the health status of the older people and enhance their life satisfaction, it is necessary to create a good family atmosphere, attach importance to family care, give the older people material and economic support, and conduct regular online and offline communication activities with them, giving full play to Internet technology, so as to encourage them to establish a positive psychological state and maintain their physical and mental health.

Behavioral independence plays an important intermediary role in the relationship between the perceived importance of the Internet and the older people's life satisfaction/self-rated health. With the increase of age, an individual's function gradually declines, which may be accompanied by obstacles that greatly affect their life satisfaction and health status (44, 45). At present, with the development of science and technology, the traditional lifestyle has changed, and IC cards, intelligent washing machines, and integrated kitchen and bathroom facilities have all provided enhancement to daily life (46, 47). However, older people often lack the ability to deal with new things, and the question of how to improve their behavioral independence has become an important issue (48). Studies have increasingly shown that learning the requisite skills to use smart devices, engage in outdoor activities, and use the Internet can improve older people's behavioral independence. For example, the use of smart wheelchairs and smart glasses can enhance older people's mobility and reading ability, and greatly improve their independence. At the same time, use of the Internet can improve the cognition level of older people with respect to new things; this can improve their independence and change their lifestyle, which plays an important role in improving their life satisfaction and health (49). In order to effectively resolve the “digital divide” experienced by the older people, the speed of development of aging-friendly construction can be improved by setting up special actions for Internet applications that are aging-friendly and barrier-free. The older people can also be prompted to adapt to the intelligence era through peer education and other methods, so as to enhance their ability to act independently, and thus improve their quality of life and health.

The study found that the life satisfaction and self-rated health status of the older people in China in 2018 were higher than in 2016. One of the reasons may be that since 2016 China has formulated the “Healthy China 2030” plan, and actively carried out educational activities for the older people covering fitness, health-care, prevention and treatment of diseases, and rehabilitation. It has also optimized the living arrangements, transportation, medical care, and nursing environment for the older people. Creating a safe, convenient, comfortable, and barrier-free livable environment for the older people has greatly improved their expected healthy life and happiness (50). In addition, research shows that the life satisfaction of the older people and their self-rated health have a continuous cross effect. Previous studies have found that life satisfaction is related to health status, but the causal relationship is not clear. The results of this study show that life satisfaction and self-rated health are cross influenced and have a certain persistence. That is, front-end self-rated health (2015–2016) has a higher impact on back-end life satisfaction (2017–2018) than front-end life satisfaction (2015–2016). The standardized coefficients are 0.09 and 0.05, respectively. At the same time, the influence of life satisfaction and self-rated health is persistent, which is consistent with previous research results (51, 53). This may be because the higher the individual's life satisfaction in the previous year, the better their material living standard, which improves future physical and mental health to a certain extent. Therefore, from the perspective of prevention, preventing diseases should be given priority in order to improve the physical and mental health of the older people, thus effectively maintaining their life satisfaction.

To sum up, the perceived importance of the Internet has an important impact on the life satisfaction and health of the older people. Moreover, family atmosphere and behavioral independence play an important mediating role in perceived importance of the Internet and life satisfaction and health of the older people. Therefore, there is a need to increase the frequency of Internet use by the older people and reduce the digital divide. In addition, in order to create a harmonious family atmosphere, children should be encouraged to guide the older people on how to use Internet devices, and help strengthen the independence of the older people, especially in terms of thought, personality, and life. Finally, life satisfaction and self-rated health of the older people have a continuous cross effect, and initiatives should start from the prevention side to improve the life satisfaction and health of the older people.

## Limitations

Some limitations to this study warrant consideration. First, the associations among perceived importance of the Internet, family atmosphere, behavioral independence, life satisfaction, and self-rated health are cross-sectional in the study and can be further validated using longitudinal data in the future. Second, since the information was gathered from the participants in the study, self-report/recall bias may have existed. However, it is not easy to achieve continued participation among cohorts of older people in a cohort study, and the sample size should not be ignored. As a result, our findings with acceptable goodness-of-fit indices deserve greater attention. Finally, our study used a Chinese family tracking survey. In the future, the CFPS data could be combined with the China Healthy Pension Tracking Survey, and qualitative case analysis of the older people's Internet use, health status, and life satisfaction could be conducted.

## Conclusions

The life satisfaction and health status of the older people should be matters of continuous concern. Life satisfaction and self-rated health have cross influences, and they are persistent on the time baseline. Family atmosphere and behavioral independence each play an important intermediary role in the older people's perception of the importance of the Internet and their life satisfaction/self-rated health. Therefore, strengthening parent–child interaction, promoting parent–child relationships, and promoting behavioral independence can effectively improve the life satisfaction and health status of the older people.

## Data availability statement

The original contributions presented in the study are included in the article/supplementary materials, further inquiries can be directed to the corresponding author/s.

## Ethics statement

This study was approved by the Ethical Review Committee of Peking University Biomedical (IRB00001052-14010), and all participants signed informed consent. The patients/participants provided their written informed consent to participate in this study.

## Author contributions

M-mJ and KG designed the study, analyzed results, drafted, revised the manuscript, and acquisition of funding. M-mJ and Z-yW drafted and revised the manuscript. KG and P-pG analyzed results and revised the manuscript. All authors read and approved the final article.

## Funding

This work was supported by the National Natural Science Foundation of China (Grant Number 72074187). The sponsors of the project had no role in the study design, data collection, data analysis, data interpretation, and writing the manuscript.

## Conflict of interest

The authors declare that the research was conducted in the absence of any commercial or financial relationships that could be construed as a potential conflict of interest.

## Publisher's note

All claims expressed in this article are solely those of the authors and do not necessarily represent those of their affiliated organizations, or those of the publisher, the editors and the reviewers. Any product that may be evaluated in this article, or claim that may be made by its manufacturer, is not guaranteed or endorsed by the publisher.

## Abbreviations

PII: perceived importance of the Internet
FA: family atmosphere
IDB: independent behavior
LF16: sense of safety in 2015–2016
SH16: self-rated health in 2015–2016
LF18: sense of safety in 2017–2018
SH18: self-rated health in 2017–2018
SD: standard deviation
SEM: structural equation modeling.
